# Influenza Virus Aerosols in Human Exhaled Breath: Particle Size, Culturability, and Effect of Surgical Masks

**DOI:** 10.1371/journal.ppat.1003205

**Published:** 2013-03-07

**Authors:** Donald K. Milton, M. Patricia Fabian, Benjamin J. Cowling, Michael L. Grantham, James J. McDevitt

**Affiliations:** 1 Maryland Institute for Applied Environmental Health, University of Maryland School of Public Health, College Park, Maryland, United States of America; 2 Department of Environmental Health, Harvard School of Public Health, Boston, Massachusetts, United States of America; 3 Department of Environmental Health, Boston University School of Public Health, Boston, Massachusetts, United States of America; 4 Department of Community Medicine and School of Public Health, Li KaShing Faculty of Medicine, The University of Hong Kong, Hong Kong, China; Erasmus Medical Center, Netherlands

## Abstract

The CDC recommends that healthcare settings provide influenza patients with facemasks as a means of reducing transmission to staff and other patients, and a recent report suggested that surgical masks can capture influenza virus in large droplet spray. However, there is minimal data on influenza virus aerosol shedding, the infectiousness of exhaled aerosols, and none on the impact of facemasks on viral aerosol shedding from patients with seasonal influenza.

We collected samples of exhaled particles (one with and one without a facemask) in two size fractions (“coarse”>5 µm, “fine”≤5 µm) from 37 volunteers within 5 days of seasonal influenza onset, measured viral copy number using quantitative RT-PCR, and tested the fine-particle fraction for culturable virus.

Fine particles contained 8.8 (95% CI 4.1 to 19) fold more viral copies than did coarse particles. Surgical masks reduced viral copy numbers in the fine fraction by 2.8 fold (95% CI 1.5 to 5.2) and in the coarse fraction by 25 fold (95% CI 3.5 to 180). Overall, masks produced a 3.4 fold (95% CI 1.8 to 6.3) reduction in viral aerosol shedding. Correlations between nasopharyngeal swab and the aerosol fraction copy numbers were weak (r = 0.17, coarse; r = 0.29, fine fraction). Copy numbers in exhaled breath declined rapidly with day after onset of illness. Two subjects with the highest copy numbers gave culture positive fine particle samples.

Surgical masks worn by patients reduce aerosols shedding of virus. The abundance of viral copies in fine particle aerosols and evidence for their infectiousness suggests an important role in seasonal influenza transmission. Monitoring exhaled virus aerosols will be important for validation of experimental transmission studies in humans.

## Author Summary

The relative importance of direct and indirect contact, large droplet spray, and aerosols as modes of influenza transmission is not known but is important in devising effective interventions. Surgical facemasks worn by patients are recommended by the CDC as a means of reducing the spread of influenza in healthcare facilities. We sought to determine the total number of viral RNA copies present in exhaled breath and cough aerosols, whether the RNA copies in fine particle aerosols represent infectious virus, and whether surgical facemasks reduce the amount of virus shed into aerosols by people infected with seasonal influenza viruses. We found that total viral copies detected by molecular methods were 8.8 times more numerous in fine (≤5 µm) than in coarse (>5 µm) aerosol particles and that the fine particles from cases with the highest total number of viral RNA copies contained infectious virus. Surgical masks reduced the overall number of RNA copies by 3.4 fold. These results suggest an important role for aerosols in transmission of influenza virus and that surgical facemasks worn by infected persons are potentially an effective means of limiting the spread of influenza.

## Introduction

Transmission of influenza virus between humans may occur by three routes: (1) direct or indirect contact between an infected and a susceptible person, usually resulting in contamination of a susceptible person's hands followed by hand to respiratory mucosa contact; (2) large droplet spray wherein droplets of respiratory fluid greater than approximately 100 µm in diameter are expelled with sufficient momentum to deliver a direct hit on the respiratory mucosa; and (3) aerosols generated by release of smaller, virus-containing droplets, as may occur during tidal breathing and coughing [1], [2], that rapidly evaporate into residual particles (droplet nuclei),which are inhaled and deposited in the respiratory tract [3]–[6]. There is significant evidence for each of these routes [7], [8], but their relative importance is not known [3]. As a result, the Institute of Medicine recommended that healthcare workers in contact with 2009-H1N1 patients use protection against all of the possible routes of infection, including use of fit-tested N95 respirators [3]. A year after the 2009 pandemic, there was no greater clarity on the importance of the various modes of transmission [9].

The U.S. Centers for Disease Control and Prevention recently funded an experimental study of person-to-person transmission to address this important knowledge gap [10]. However, an experimental study using intranasal inoculation to infect experimental donors [11] will need to show that the donors and naturally infected persons shed similar virus aerosols with regard to quantity, particle size distribution, and infectiousness, given that earlier experiments suggested that intranasal inoculation requires quantitatively larger doses and produces qualitatively milder illness than does inoculation via aerosol [12].

In an occupational hygiene context, personal protection is usually the last resort, after source mitigation and environmental controls are exhausted [13]. Thus, it is worthwhile considering whether surgical facemasks could be effective as a means of source control. The CDC recommends that persons with influenza wear surgical masks when in contact with susceptible individuals [14], [15]. However, there is only one report studying mask impact on containment of infectious large droplet spray during influenza infection [16], and no data on surgical mask impact on release of infectious viral aerosols.

In the current study of patients infected with seasonal influenza, we describe the number of copies of viral RNA in two aerosol size fractions, report the culturability of virus in the fine-particle fraction, and the effect of surgical masks.

## Results

We screened 89 volunteers: 33 (37%) tested positive for influenza using the rapid test (20 influenza A and 13 influenza B) and were asked to provide exhaled breath samples. Eight additional volunteers with negative rapid tests who reported a cough and who had a temperature of ≥37.8°C were also invited to participate. In total, 38 volunteers were confirmed to have influenza virus infection by PCR of nasopharyngeal specimens. Exhaled breath data with and without a surgical mask are complete for 37 of the 38 volunteers (21 influenza A, 16 influenza B); data for one volunteer has been excluded due to laboratory error in sample processing. One of the infected subjects reported receiving influenza vaccine for the current year. None of the subjects sneezed during the sample collection. Table 1 shows the sex, symptom and fever prevalence, and influenza virus type and Table 2 shows descriptive statistics for age and viral RNA copy number in swabs and exhaled aerosol fractions of the 37 volunteers with confirmed influenza infection. The viral copy numbers in each of the five specimens for all 37 cases are shown in Table S1.

**Table 1:** Participant's sex, symptoms, temperature, and influenza virus type.

	N	Percent
Number with complete data	37	100
Male	30	81
On antiviral medicine^a^	0	0
Asthmatic^a^	5	14
Flu shot this season^a^	1	3
Flu shot previous seasons^a^	12	32
Current smoker^a^	9	24
Tachypnea^a^	13	35
Breathing difficulty^a^	16	43
Lymphadenopathy^a^	18	49
Feverish^a^	19	51
Temperature^b^ ≥37.8°C	10	27
Type A	21	57

a Self-reported.

b At time of exhaled breath measurement.

**Table 2:** Descriptive statistics.

	Percentiles				
	Min	25^th^	Median	75^th^	Max
Age	18	18	19	20	54
Days since onset^a^	0	1	2	3	5
Nasopharyngeal swab copy number	1.7×10^3^	8.3×10^4^	4.2×10^5^	1.8×10^6^	3.4×10^7^
Coarse particle copy number with mask	0	0	0	0	7.7×10^1^
Coarse particle copy number no mask	0	0	0	3.7×10^1^	2.9×10^4^
Fine particle copy number with mask	0	5	2.2×10^1^	2.5×10^2^	2.4×10^4^
Fine particle copy number no mask	0	1.1×10^1^	1.1×10^2^	5.6×10^2^	1.3×10^5^

a **At time of exhaled breath measurement.**

**Figure 1:**
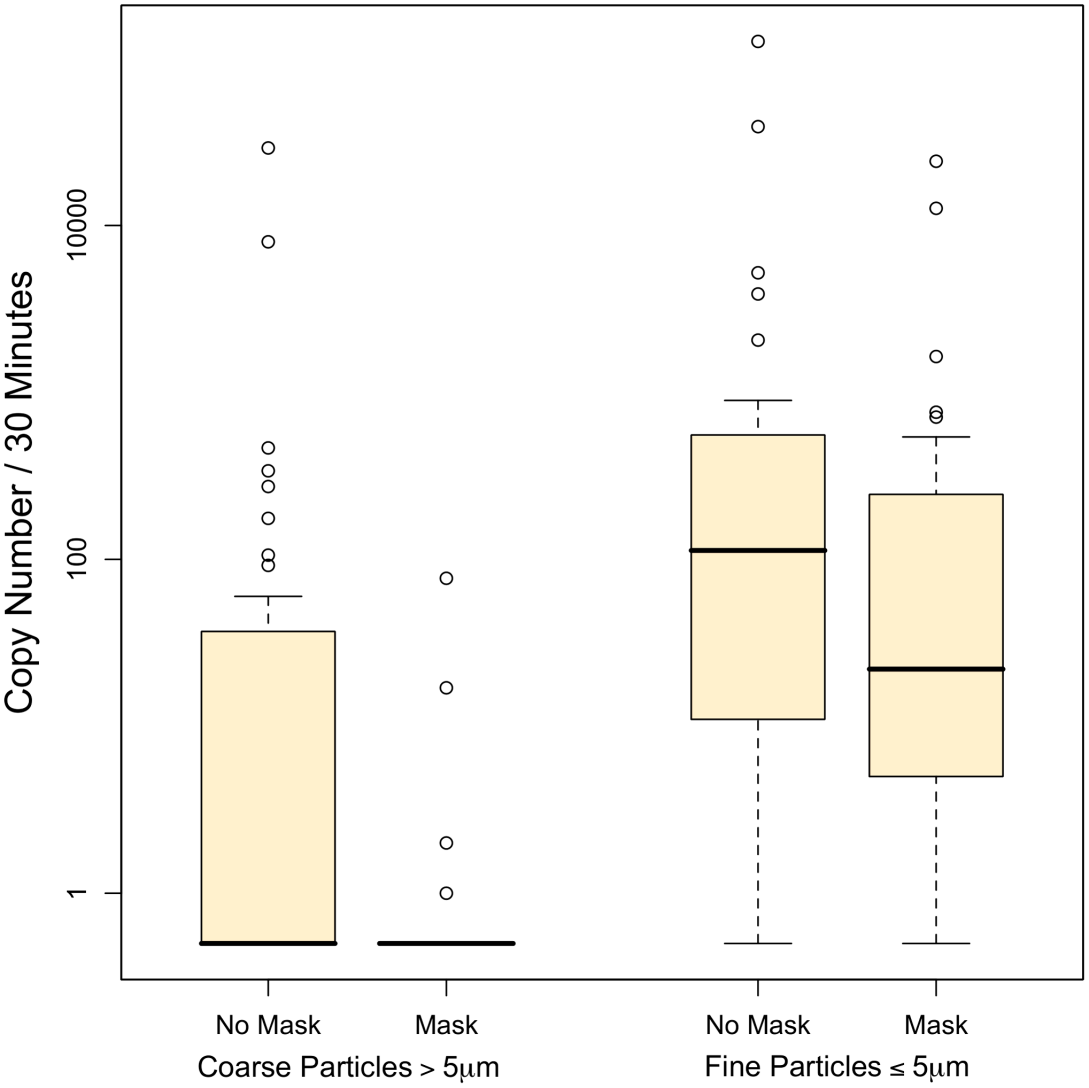
Influenza virus copy number in aerosol particles exhaled by patients with and without wearing of an ear-loop surgical mask. Counts below the limit of detection are represented as 0.5 on the log scale.

We detected influenza virus RNA in the coarse fraction (particles greater than 5 µm) collected from 11% (4 of 37 volunteers) while wearing surgical masks and from 43% (16 of 37) while not wearing a mask (relative risk for virus detection with mask = 0.25, 95% confidence interval (CI) 0.09 to 0.67; McNemar's test p = 0.003). The median number of coarse fraction viral copies (Figure 1) was below the limit of detection with and without facemasks; the 75^th^ percentile dropped from 37 to below the limit of detection with use of surgical masks. Using Tobit analysis, we estimated that the geometric mean coarse fraction copy number without a facemask was 12 (95% confidence interval (CI), 4 to 37) and that the effect of facemasks was to produce a statistically significant 25 fold reduction in the copy number (95% CI 3.5 to 180, p = 0.002) to <0.5 copies per 30 min sample.

We detected viral RNA in 78% (29 of 37) of fine particle samples collected from volunteers when they were wearing a mask and in 92% (34 of 37) of samples collected when they were not wearing a mask. Thus, the relative risk for any virus detection with mask versus without a mask was 0.85 and borderline statistically significant (CI 0.72 to 1.01; McNemar's test p = 0.06). However, the reduction in copy number was statistically significant: The median number of viral copies in the fine particle fraction was 250 with masks and 560 without masks. The geometric mean copy number in the fine particle fraction without a facemask was 110 (95% CI 45 to 260) and the facemasks produced a 2.8 fold reduction in copy number (95% CI 1.5 to 5.2, p = 0.001).

Combining the coarse and fine fractions, we detected viral RNA in 29 (78%) subjects when wearing facemasks and 35 (95%) when not wearing facemasks (McNemar's test p = 0.01). Surgical masks produced a 3.4 (95% CI 1.8 to 6.3) fold reduction in viral copies in exhaled breath.

Fine fraction copy numbers were on average 8.8 (95% CI 4.1 to 19) times larger than coarse fraction copy numbers. The coarse and fine fraction copy numbers were correlated (r = 0.60, p<0.0001). The viral load in the nasopharyngeal swab specimen, however, was not correlated with that in the coarse fraction (r = 0.17, p = 0.31) and only weakly with that in the fine fraction (r = 0.29, p = 0.08). There was no significant difference in copy number between influenza A and B virus infection in either the coarse (p = 0.28) or fine (p = 0.26) fraction. Reported asthma (p = 0.029) and feverishness (p = 0.014) were associated with significantly lower fine fraction copy numbers. However, coarse fraction copy numbers were not significantly impacted and temperature measured at the time of testing was not associated with exhaled copy numbers. Vaccination in any prior year was associated with a non-significant trend toward lower copy numbers in coarse (p = 0.11) and fine fractions (p = 0.15); there were too few having received the current season's vaccine to analyze. Self-reported tachypnea, breathing difficulty, smoking, and lymphadenopathy were not associated with significant shifts in exhaled copy numbers.

We recovered infectious virus from fine particle samples (with and without mask) produced by the two subjects with the highest numbers of viral RNA copies in the fine particle fraction after blind passage on MDCK cells. Sequence analysis showed that the two isolates were seasonal H1N1, with sequence differences from each other and unrelated to any viruses present in the Veterinary Medicine laboratories at the time these samples were cultured.

Virus copy number (Table 3) declined with time since onset of symptoms. In the coarse fraction, each additional day after onset was associated with a 6.0 fold drop in the number of virus copies detected (95% CI 1.7 to 21 fold). Fine particles also declined with time, each additional day after onset was associated with a 2.4 fold drop in the number of copies detected (95% CI 1.1 to 5.1 fold).

**Table 3:** Copy number coarse and fine exhaled particles without surgical mask by day since onset of influenza symptoms.

			Number of Virus Particles		
Days Since Onset^a^	Particle Size	Number of Cases	Min	Median	Maximum
1	Swab	10	2.1×10^4^	1.1×10^6^	3.4×10^7^
	Coarse		<LD	2.3×10^1^	2.9×10^4^
	Fine		4	6.1×10^2^	1.3×10^5^
2	Swab	15	1.7×10^4^	1.0×10^5^	3.4×10^6^
	Coarse		<LD	<LD	4.7×10^2^
	Fine		<LD	2.1×10^1^	3.9×10^4^
3	Swab	7	2.3×10^4^	1.4×10^6^	1.0×10^7^
	Coarse		<LD	<LD	1.1×10^2^
	Fine		2	3.7×10^1^	5.3×10^2^
4	Swab	3	8.1×10^4^	4.2×10^5^	1.5×10^6^
	Coarse		<LD	<LD	<LD
	Fine		3.2×10^1^	7.5×10^1^	4.4×10^2^

a Because there were only single cases studied on day 0 (day of onset) and on day 5 since onset of symptoms, only data for cases studied on days 1 through 4 after onset of symptoms are shown.

## Discussion

We measured exhaled influenza viral particle copy number by quantitative RT- PCR in two particle size fractions, ≥5 µm (coarse) and <5 µm (fine), and assayed the fine fraction for culturable virus. We observed that viral copy numbers were greater in the fine than in the coarse fraction, and recovered infectious virus from the fine particle fraction collected from the two samples with the highest RNA copy numbers. These results, combined with older data suggesting that the infectious dose via aerosol is about two orders of magnitude lower than via large droplets [12], suggest an important role for aerosols in seasonal influenza transmission.

Surgical masks nearly eliminated viral RNA detection in the coarse aerosol fraction with a 25 fold reduction in the number of viral copies, a statistically significant 2.8 fold reduction in copies detected in the fine aerosol fraction, and an overall statistically significant 3.4 fold reduction of viral copy number in the exhaled aerosols. This finding supports current Centers for Disease Control and Prevention recommendations that healthcare facilities encourage patients with influenza-like illness to don surgical facemasks as one component of an influenza infection control program [17].

When volunteers were not wearing surgical masks, we detected virus RNA in coarse particles exhaled by 43% and in fine particles exhaled by 92% of influenza patients. This is in contrast to the report by Johnson et al [16], who detected influenza virus RNA in cough generated large droplet spray from 100% of influenza patients over two brief sampling trials, and from 78% on each trial. These discrepant findings are likely due to the very different collection techniques and particle sizes collected in these two studies. We used a specially designed aerosol sampler to collect particles from 0.05 to 50 µm in diameter. Johnson et al, by contrast, used simple deposition on petri dishes, and based on particle settling rates and collection times, that method would have been unlikely to collect particles with diameters of less than approximately 50 µm because smaller particles would have remained suspended in air and flowed around the petri dishes.

We view results from Johnson et al and the present study as complementary. Together the studies show that surgical masks can limit the emission of large droplet spray and aerosol droplets larger than 5 µm [16]. However, surgical masks are not as efficient at preventing release of very small particles. It is well known that surgical masks are not effective for preventing exposure to fine particles when worn as personal protection [18]. We had hypothesized that when used as source control, exhaled droplets might be large enough prior to evaporation to be effectively captured, primarily through impaction. This appears to be true for virus carried in coarse particles. But the majority of virus in the exhaled aerosol appear to be in the fine fraction that is not well contained. Nevertheless, the overall 3.4 fold reduction in aerosol copy numbers we observed combined with a nearly complete elimination of large droplet spray demonstrated by Johnson et al. suggests that surgical masks worn by infected persons could have a clinically significant impact on transmission. For example if one hypothesized that all transmission were due to aerosol particles <50 µm, and estimated a reproductive number of 1.5 for influenza (i.e. each infection generates 1.5 new infections on average at the start of the epidemic) [19], then the use of surgical masks by every infected case could reduce the reproductive number below 1 [20]. Compliance, however, would be a major limitation resulting in lower efficacy in real-world practice [21], [22].

While it is generally assumed that large droplets shed from the respiratory tract contain infectious virus, there are limited data that indicate that fine particle aerosols released from the human respiratory tract contain infectious virus. In one previous study by Lindsley et al, infectious virus was detected in 2 of 21 cough aerosol samples, once with a sampler that did not discriminate between coarse and fine particles and once in the coarse particle fraction of a second instrument [23]. This observation, along with our observation that it was possible to recover culturable virus from the fine-particle fraction using our device demonstrates that humans generate infectious influenza aerosols in both coarse and fine particle fractions. This lends support to the hypothesis that aerosols may be a common pathway for influenza transmission among humans [8], [24]. However, a clear test of the hypothesis requires intervention studies that can interrupt only one mode of transmission without interfering with others [25].

We only detected infectious virus in exhaled breath samples with high (10^4^ to 10^5^) copy numbers by quantitative RT-PCR. This implies that the ratio of total viral particles to infectious virus was about 10^3^ to 10^4^, compared with 10^2^ to 10^3^ for laboratory stocks and experimental aerosols [26]. It is not yet known whether the low recovery of infectious virus (despite high copy numbers of viral RNA) represents technical difficulties in sampling and culturing exhaled breath samples or whether the vast majority of the virus exhaled by influenza A patients is actually non-infectious. These findings are consistent with those by Lindsley et al. [23] We designed the sampler specifically to overcome problems with existing bioaerosol samplers, including efficiently collecting sub-micron particles into a liquid and use of appropriate buffer to preserve infectiousness [27]. We have previously shown that collection on solid, dry collection media resulted in large losses of culturability [26]. Therefore, we did not attempt to culture the coarse fraction collected on a Teflon substrate. Subsequent studies in our laboratory indicated that about 50% of the infectious virus is lost during the concentration step of our procedure (data not shown), suggesting that this is one contributing factor in the low rate of recovery of infectious virus in this study.

The lack of strong correlation between the viral load in the nasopharyngeal and aerosol samples is possibly of interest. This may merely be a result of nasopharyngeal sample variability; in future studies, control for sample quality by PCR of a cellular gene may be helpful. Our sampler, as is the case with all samplers for fine and ultrafine particles, has an upper limit to the size droplet that can be pulled into its inlet airstream. Thus, a second possible explanation for the lack of correlation is that the nasopharynx is primarily a source for very large droplets (>50 µm) that we would not have detected. Furthermore, none of our subjects sneezed; an efficient method of generating droplets from the upper respiratory tract. This may imply that the smaller droplets we detected were generated in the lower respiratory tract and that the viral load at that location is not strongly correlated with the nasopharyngeal load. Alternatively, shedding into aerosol droplets may be driven by other host factors (e.g. asthma, symptom severity, and immune response), co-infection with other agents, virus factors affecting release from the epithelium, or the nature of the resident microbiome. If shedding into aerosol is determined in large part by the location of infection in the respiratory tract, this may have implications for experimental studies of transmission [11], [28]. Such studies will need to monitor aerosol shedding to determine whether nasal inoculation of donors results in aerosol shedding that mimics naturally acquired infection to validate the experimental design and aid the interpretation of results.

Most of the viral aerosol generation we observed occurred during the first days of symptomatic illness (Table 3), consistent with studies of shedding monitored by nasal washes [29]. We studied each individual on only one occasion and, by design, have little data beyond day 3. Further longitudinal studies of viral aerosol generation are needed to confirm these findings. New study designs will be needed to examine aerosol generation before and on the day of symptom onset in community acquired infection. A limitation of our study is that we recruited patients with certain signs and symptoms or who were positive on a rapid test or had fever, and therefore our data could be biased towards patients with higher viral loads [21]. However, we still observed significant inter-individual variation and modeling suggests that cases with higher viral loads are disproportionately important in the spread of influenza [30], [31]. Additional studies are also needed to determine how aerosol generation correlates with symptoms (including milder disease), presence of other health conditions, age (we studied a narrow age distribution), and co-infection with other respiratory viruses so that recommendations for infection control can be critically evaluated.

## Methods

### Patient population

We recruited volunteers with influenza-like illness from the Lowell, MA community, primarily among students and staff of the University of Massachusetts, beginning January 29 and ending March 12, 2009. The study protocol was approved by the Institutional Review Boards of the University of Massachusetts Lowell, Lowell General Hospital, and Saints Memorial Hospital, Lowell, MA. Oral informed consent was obtained by providing each subject with a detailed consent information form. Collection of a signed copy of the form was waived because it would have been the only personally identifiable information retained by this minimal risk study.

Volunteers learned of the study through flyers and notices posted on campus and by referral from health care providers. We screened self-referred volunteers by telephone for influenza-like illness (ILI). Persons who reported onset of fever and cough within the preceding 72 hours or were referred by a health care provider were invited to the laboratory for testing. We collected a nasopharyngeal specimen using a flocked swab (501CS01, Copan Diagnostics, Murrieta, CA) and temperature was taken with a digital ear thermometer (Model 18-200-000, Mabis Healthcare, Waukegan, IL). All volunteers with a temperature ≥37.8°C and a cough and volunteers without fever who provided a nasopharyngeal specimen positive for influenza by point of care testing (QuikVue Influenza A/B, Quidel Corp., San Diego, CA) were invited to provide exhaled breath samples, answer a questionnaire, and provide a second nasopharyngeal specimen for analysis by PCR. Only subjects with influenza infection confirmed by PCR were included in the data analysis.

### Exhaled breath collection

**Figure 2:**
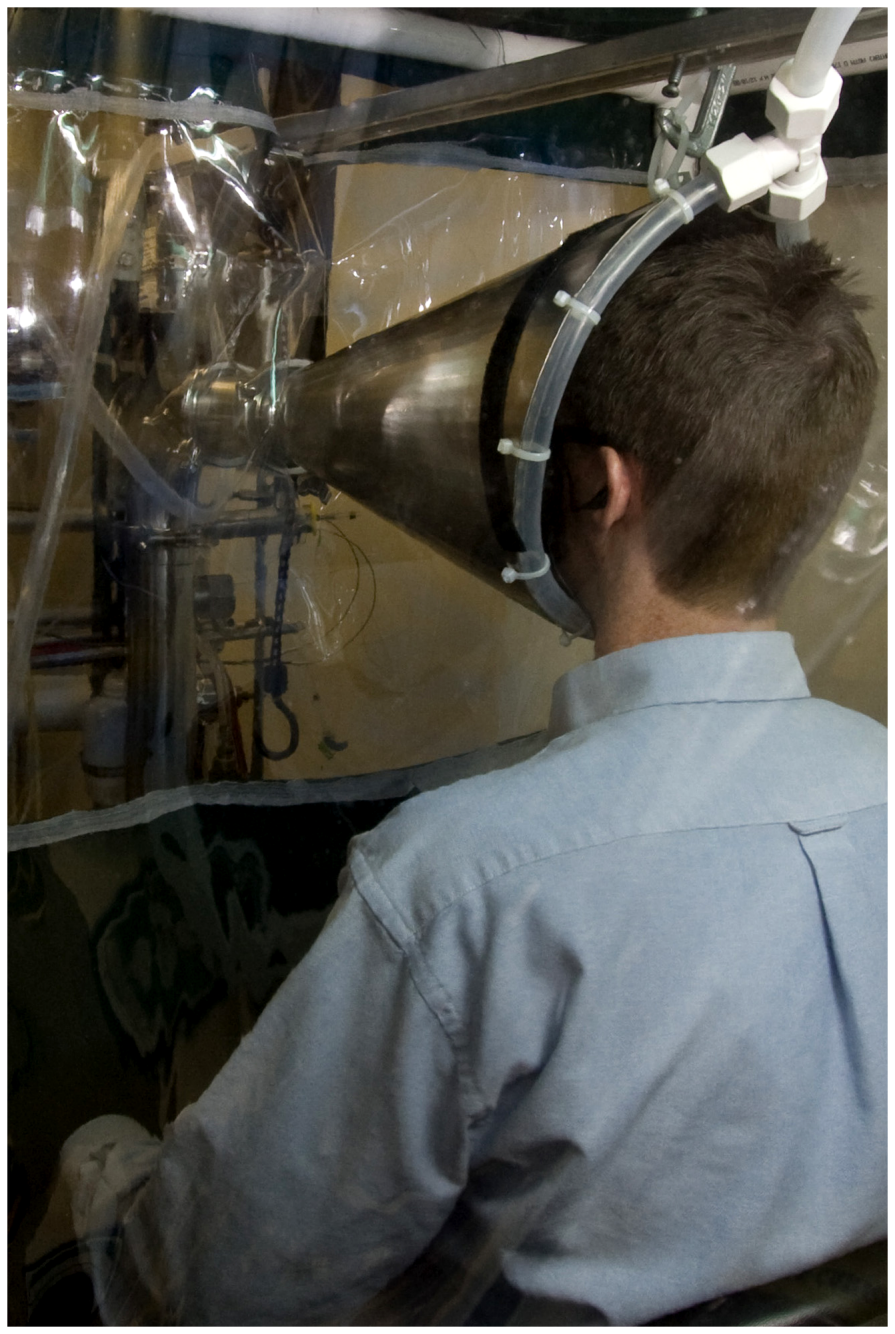
Exhaled breath collection system. Each volunteer sat as shown with face inside the inlet cone of the human exhaled breath air sampler inside a booth supplied with HEPA filtered, humidified air for 30 min while wearing an ear-loop surgical mask. Three times during the 30 min each subject was asked to cough 10 times. After investigators changed the collection media, the volunteer sat in the cone again, without wearing a surgical mask, for another 30 min with coughing as before.

We collected exhaled breath with the subject seated in front of the inlet for a sampler designed for human exhaled breath collection, Figure 2, (G-II) described in detail by McDevitt et al. [27] Briefly, the G-II inlet was cone shaped so that the subject's face was situated inside the large end of an open cone with air drawn continuously around the subject and into the sampler. The cone allows the subject to breathe normally and unlike use of a mouthpiece, the subject could also wear a mask. The cone served as a capture type ventilation hood allowing collection of exhaled breath with minimal fugitive emissions even when the subject was wearing a mask with resultant redirection of flow. Intake air (130 L/min) flowed through a conventional slit impactor that collected particles larger than 5 µm on a Teflon surface (“coarse” particle fraction). To collect a “fine” particle fraction, water vapor was condensed on the remaining particles, which created droplets large enough to be captured by a 1.0-µm slit impactor. The 1.0-µm impactor was composed of a slit and a steel impaction surface sealed inside a large reservoir. Impacted droplets drained from the impaction surface into a buffer-containing liquid in the bottom of the reservoir. Concentrated buffer was pumped into the reservoir during collection to match the accumulation of water from collected droplets and maintain phosphate buffered saline with 0.1% bovine serum albumin throughout collection. The sampler was shown to be 85% efficient for particles greater than 50 nm in diameter and was comparable to the SKC BioSampler for detection and recovery of influenza A/PR/8/34 H1N1 by PCR and culture. Between subjects, the apparatus was disassembled and cleaned with a 0.5% hypochlorite solution.

Exhaled particles were collected for 30 minutes while the subject wore an ear-loop surgical mask (Kimberly-Clark, Roswell, GA) and then for 30 minutes without a mask. Subjects were asked to cough 10 times at approximately 10-minute intervals for a total of 30 coughs during each 30 minute sample. One subject coughed frequently such that forced coughs were not required. No subjects were observed to sneeze.

### Sample analysis

Immediately after collection, the Teflon impaction surface was removed and temporarily stored at −20°C. The impactors were scraped with a flocked swab wetted with Dulbecco's phosphate buffered saline with calcium and magnesium (Hyclone, Thermo Scientific, Waltham, MA) with 0.1% bovine serum albumin (DPBS++BSA). The swab was eluted in 600 µl of DPBS++BSA for 1 minute with vortexing. The resulting sample was stored at −80°C.

The fine particle fraction collected in DPBS++BSA buffer (100 to 150 ml volume) was maintained at 4°C and concentrated by ultrafiltration using Amicon Ultra 15 filter units with a molecular weight cut off of 100 kD (Millipore, Bedford, MA) to a volume of approximately 400 µl. Following ultrafiltration, the filter was washed with 200 µl of DPBS++BSA, and the wash solution was combined with the retentate. Samples were stored at −80°C.

RNA extraction in Trizol-chloroform, reverse transcription, and quantitative PCR were performed as previously described [1], [32]. Quantitative PCR was performed using an Applied Biosystems Prism 7300 detection system (Foster City, CA) for coarse fraction samples or a LightCycler 480 (Roche, Indianapolis, IN) for the fine particle fraction. Duplicate samples were analyzed using influenza A and B primers described by van Elden et al. [33] A standard curve was constructed in each assay with cDNA extracted from a stock of influenza A (A/Puerto Rico/8/1934, Advanced Biotechnologies Incorporated, Columbia, MD) with a concentration of 3.0×10^11^ virus particles per mL or a stock of influenza B (B/Lee/1940, Advanced Biotechnologies Incorporated, Columbia, MD) with a concentration of 8.6×10^10^ virus particles per mL as determined by electron microscopy. Results are expressed as the total number of virus particles by reference to the standard curve, rounded to the closest integer value. The limits of detection were 6 and 11 viral RNA copies per qPCR well for influenza A and B respectively. Fine particle samples from all subjects were cultured for infectious virus on MDCK cells. Confluent cells in 24-well plates (Corning, NY, USA)were inoculated with 0.1 ml of the concentrated sample diluted 1∶1 in OptiMEM® I medium (Invitrogen, Carlsbad, California). The plates were incubated at 37°C for 1 h with rocking every 15 min, and 0.8 ml of OptiMEM® I media with 1 µg/ml of TPCK-trypsin was added to each well and incubated for 72–96 h. The cells were checked daily for cytopathic effect (CPE) and if none was detected, two blind passages were performed using cell supernatant. At each passage, supernatants were tested for influenza virus by hemagglutination (HA) assay using 0.5% chicken red blood cells. Positive samples were confirmed by Flu DETECT (Synbiotics, CA, USA) strip test and by amplification of the hemagglutination (HA) gene by RT-PCR followed by sequencing.

### Statistical analysis

We analyzed the effect of surgical masks as a) log relative risk for production of any virus aerosols assuming a binomial distribution using generalized estimating equations with exchangeable within-subject correlation to account for repeated measures, and b) the geometric mean counts of virus particles detected in exhaled breath by qPCR and fractional reduction in copy number using Tobit regression analysis on log copy number with a random effect to account for variability between individuals. Tobit analysis was also used to compare coarse and fine particle fractions. Tobit regression avoids bias that would arise from assigning samples below the limit of detection a specific value such as zero or the limit divided by the square root of 2. Surgical mask use was the dependent variable. We also computed McNemar's test for paired samples to examine mask effect and Spearman's correlation coefficient to examine the relationship between the load in the nasopharyngeal swab and aerosol fractions. Statistical analyses were performed using SAS (Procs GenMod, NLMixed, Lifereg, Freq, Corr, and Means, version 9.2, Cary, NC).

## Supporting Information

Table S1 Copy number and influenza type in five assayed samples per subject.
(DOCX)
