# Supplementary material for: Influenza Virus Aerosols in Human Exhaled Breath: Particle Size, Culturability, and Effect of Surgical Masks
Source: PLoS Pathog. 2013 Mar 7;9(3):e1003205. doi: 10.1371/journal.ppat.1003205 (PMC3591312; doi:10.1371/journal.ppat.1003205)
Supplement: Table S1 — Copy number and influenza type in five assayed samples per subject. (DOCX) [file ppat.1003205.s001.docx]

**Table S1. Copy number and influenza type in five assayed samples per subject.**

| **Obs** | **Mask_Fine** | **No_Mask_Fine** | **Nasal_Swab** | **Mask_Coarse** | **No_Mask_Coarse** | **PCR_type** |
| --- | --- | --- | --- | --- | --- | --- |
| **1** | 16 | 4 | 1377090 | 0 | 0 | A |
| **2** | 7 | 37 | 1523075 | 0 | 176 | A |
| **3** | 7 | 13 | 1725 | 0 | 92 | A |
| **4** | 38 | 154 | 8545 | 1 | 17 | A |
| **5** | 30 | 181 | 101805 | 0 | 7 | A |
| **6** | 30 | 34 | 627610 | 0 | 7 | A |
| **7** | 24239 | 126587 | 17550000 | 0 | 29118 | A |
| **8** | 3 | 0 | 1455730 | 0 | 3 | A |
| **9** | 294 | 10 | 62445 | 17 | 0 | A |
| **10** | 5 | 533 | 9300000 | 0 | 0 | B |
| **11** | 12665 | 39087 | 3335010 | 2 | 465 | A |
| **12** | 144 | 37 | 10200000 | 77 | 0 | A |
| **13** | 0 | 4 | 270635 | 0 | 0 | A |
| **14** | 0 | 2 | 2980495 | 0 | 0 | A |
| **15** | 24 | 21 | 102972 | 0 | 0 | A |
| **16** | 0 | 0 | 5715 | 0 | 0 | A |
| **17** | 78 | 433 | 1102606 | 0 | 0 | A |
| **18** | 22 | 479 | 255097 | 0 | 0 | B |
| **19** | 1640 | 2057 | 1147719 | 0 | 7980 | A |
| **20** | 15 | 8 | 1820604 | 0 | 0 | A |
| **21** | 0 | 0 | 38204 | 0 | 0 | A |
| **22** | 0 | 7 | 1833087 | 0 | 0 | B |
| **23** | 541 | 3888 | 2092702 | 0 | 273 | A |
| **24** | 8 | 895 | 159772 | 0 | 0 | B |
| **25** | 245 | 672 | 25450000 | 0 | 37 | B |
| **26** | 10 | 32 | 418082 | 0 | 0 | B |
| **27** | 454 | 787 | 82771 | 0 | 60 | A |
| **28** | 0 | 11 | 21006 | 0 | 0 | B |
| **29** | 761 | 230 | 23600 | 0 | 106 | B |
| **30** | 319 | 666 | 110196 | 0 | 14 | B |
| **31** | 181 | 556 | 33750000 | 0 | 31 | B |
| **32** | 711 | 5206 | 298061 | 0 | 339 | B |
| **33** | 54 | 217 | 1069609 | 0 | 0 | B |
| **34** | 0 | 113 | 419600 | 0 | 0 | B |
| **35** | 8 | 443 | 80575 | 0 | 0 | B |
| **36** | 9 | 19 | 6340 | 0 | 0 | B |
| **37** | 0 | 75 | 1526445 | 0 | 0 | B |
